# Acute appendicitis and retroperitoneal abscess: rare complications of sigmoid diverticulitis

**DOI:** 10.1016/j.radcr.2021.04.075

**Published:** 2021-06-08

**Authors:** Richard L. Hesketh, Michelle Fong, Sophie Shepherd, Venugopala Kalidindi

**Affiliations:** a Department of Radiology, North Middlesex University Hospital, Sterling Way, London, N18 1QX, UK; b Department of Surgery, North Middlesex University Hospital, Sterling Way, London, N18 1QX, UK; c Department of Clinical Radiology, University College London Hospital, 235 Euston Road, London, NW12BU, UK

**Keywords:** Diverticulitis, Appendicitis, Retroperitoneal abscess, Interventional radiology, Acute abdomen, CT

## Abstract

Diverticulitis is a common cause of an acute surgical abdomen and computed tomography has become an essential part of work up particularly to identify complications that commonly include intraperitoneal perforation, abscess and fistula formation. We report the case of an 81-year-old male who presented to the emergency department with acute lower abdominal pain and was found to have sigmoid diverticulitis with the rare complications of a diverticular abscess that had formed a sinus tract and perforated into the retroperitoneum and secondary acute appendicitis. Initial management was with intravenous antibiotics, a Hartmann's procedure and appendicectomy. Subsequently the retroperitoneal collection was drained percutaneously. The case was further complicated by the patient's multiple co-morbidities and unfortunately the patient died 6 weeks after admission from sepsis. This case highlights the role of computed tomography in the pre- and post-operative period to identify complications which are often clinically occult and require early surgical and interventional radiology management to optimize outcomes.

## Introduction

Diverticulitis and acute appendicitis are among the most common causes of an acute surgical abdomen, with estimated incidences of approximately 110 and 190 per 100,000, respectively [1,2]. The 2 presentations can be difficult to differentiate clinically and well documented mimics of appendicitis include right colonic diverticulitis, cecal diverticulitis or an inflamed redundant loop of sigmoid located in the right iliac fossa. Herein we describe a presentation of acute sigmoid diverticulitis with the rare intra- and extra-peritoneal complications of secondary acute appendicitis and retroperitoneal perforation and abscess.

## Case report

### Clinical presentation

An 81-year-old male presented to the emergency department with 4 days of abdominal pain and anorexia followed by a fall and 14 h long lie on the floor. He denied any nausea, vomiting or change in bowel habit. The patient had multiple co-morbidities including stage IV chronic kidney disease secondary to polycystic kidney disease, ischemic heart disease, atrial fibrillation, type 2 diabetes, hypothyroidism, erosive gastritis, obesity and diverticular disease. On examination the patient was peripherally shutdown, had poor bibasal air entry and a peritonitic abdomen with left flank fullness. The patient was tachycardic (125 bpm), hypotensive (100/55 mm Hg), tachypnoeic (36 bpm) and afebrile (37°C), with initial blood results supporting a diagnosis of severe sepsis on a background of chronic kidney disease (Table 1).

**Table 1:** Admission laboratory results.

Lab parameter	Normal range	Admission result
White cell count (× 10^9^/L)	6- 11	25.2
C-reactive protein (mg/mL)	<5	440
Ph	7.35- 7.45	7.27
Lactate (mmol/L)	< 2	4.7
Urea (mmol/L)	2.5- 7.1	26
Creatinine (µmol/L)	< 107	275
eGFR (mL/min/1.73^2^)	> 60	19

### Imaging findings and diagnosis

**Fig. 1:**
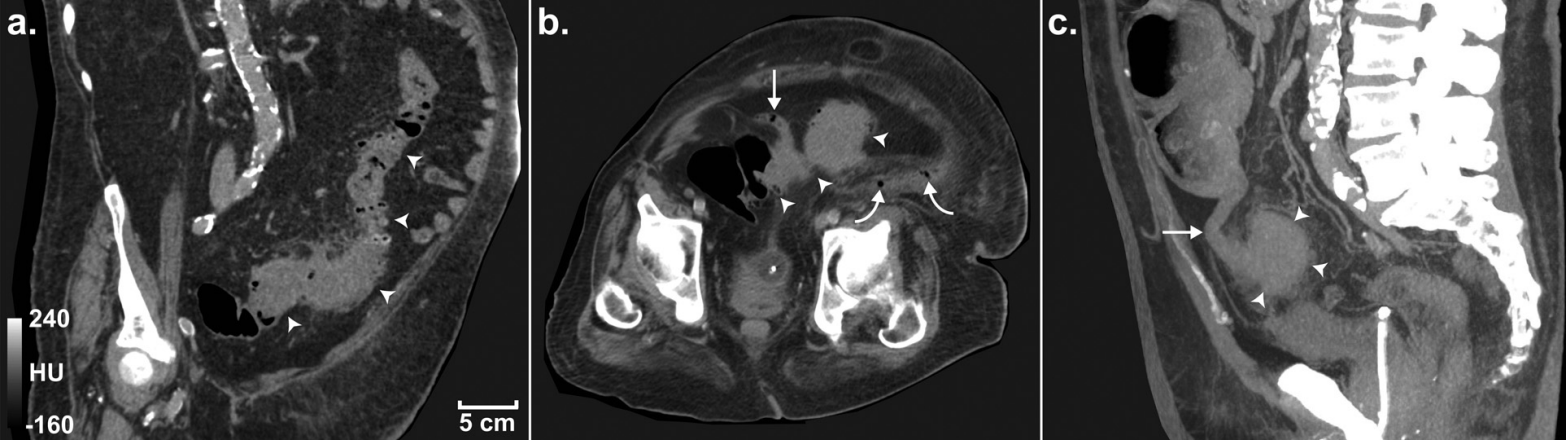
Admission CT images showing sigmoid diverticulitis, appendicitis and locules of free gas in the left retroperitoneum. (A) 1.25 mm thick coronal oblique slice demonstrating a long segment of thickened sigmoid and descending colon (arrowheads) with multiple inflamed diverticula; (B) 1.25 mm thick axial slice demonstrating the inflamed appendix (arrow) adherent to the inflamed sigmoid (arrowheads) and locules of gas within the retroperitoneal space (curved arrows) and (C) a sagittal, 30 mm thick maximum intensity projection demonstrating the position of the appendix (arrow) adherent to the sigmoid colon. All images have a window width and level of 400 and 40, respectively, and the in-plane resolution of all images is 0.63 mm.

**Fig. 2:**
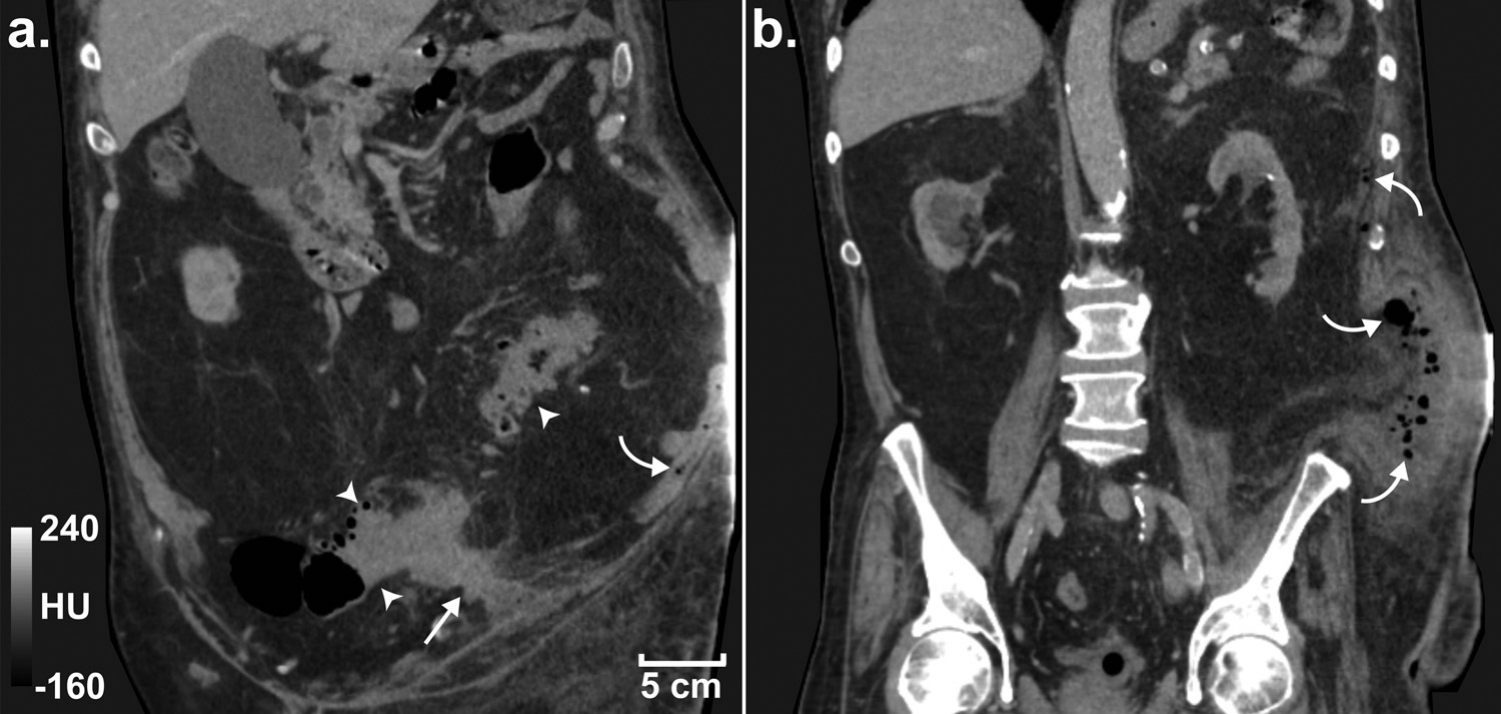
Admission CT images demonstrating the abscess forming a sinus tract between the sigmoid colon and retroperitoneum. (A) Coronal slice demonstrating sigmoid diverticulitis (arrowheads), the abscess/sinus (arrow) and locules of free gas (curved arrow) in the retroperitoneum. (B) A more posterior coronal slice demonstrating the extent of the free gas and fluid in the retroperitoneum. All images have a window width and level of 400 and 40, respectively, and a spatial resolution of 0.63 × 0.63 × 3 mm.

Portal-venous phase contrast-enhanced computed tomography (CT) of the abdomen and pelvis demonstrated an approximately 20 cm long segment of circumferentially thickened sigmoid colon containing numerous diverticula and severe pericolic inflammatory stranding (Fig. 1A). A 5 cm long diverticular abscess extended laterally from the sigmoid colon through the left lateral conal fascia with fluid and gas tracking superiorly in the posterior pararenal space to just inferior to the spleen (Fig. 2). There was no free intra-peritoneal, subcutaneous or mediastinal gas and no significant volume of free intra-peritoneal fluid. Additionally, the tip of the appendix was adherent to the inflamed sigmoid and the appendix was dilated to 12 mm in diameter with surrounding inflammatory fat stranding (Fig. 1). A diagnosis of sigmoid diverticulitis, diverticular abscess complicated by retroperitoneal perforation and acute appendicitis was made.

### Management

The pre-operative P-POSSUM score was 95.8% mortality and 99.9% morbidity. A second consultant surgical opinion was sought given his high-risk profile and, taking into account the patient's strong wishes, a joint decision was made to proceed with a laparotomy.

Intraoperative findings were an inflammatory mass attached to the left lateral sidewall containing a loop of perforated sigmoid colon and adherent appendix, cecum and distal small bowel. A sigmoid colectomy with rectal stump and end colostomy formation (Hartmann's procedure) and appendicectomy were performed. Minimal pus was found in the left lateral sidewall at time of operation and the retroperitoneum was not explored. Intra-abdominal drains were placed in the left paracolic gutter and pelvis. Histology confirmed perforated sigmoid diverticulitis with acute transmural inflammation, pericolic tissue abscess formation and acute suppurative appendicitis.

The patient was extubated on day 1 and was managed in intensive care for 8 days requiring high levels of oxygen for a co-existing pneumonia. Following stepdown to the surgical ward the patient developed worsening sepsis and CT imaging, performed on post-operative day 21, demonstrated an enlarged, complex retroperitoneal collection in the left flank. In addition to intravenous antibiotics, 2 percutaneous drains were inserted into the retroperitoneal collection but unfortunately the patient deteriorated with worsening sepsis and died on post-operative day 40.

## Discussion

We present the case of an 81-year-old man with perforated sigmoid diverticulitis complicated by acute appendicitis and retroperitoneal abscess formation. Perforation is a common complication of acute diverticulitis. The vast majority are contained perforations but in 1%–2% there is free intra-abdominal gas [3,4]. Pneumo-retroperitoneum is a far rarer occurrence that can occur secondary to perforation of the posterolateral wall of the ascending and descending colon which lacks a true mesentery [5]. Occasionally, sigmoid perforation can also result in pneumo-retroperitoneum with gas able to pass between the folds of the mesosigmoid [6,7]. However, in this instance a diverticular abscess formed a sinus between the inflamed sigmoid colon and retroperitoneal space with free gas and pus subsequently tracking into the retroperitoneum, a rarely reported phenomenon [8].

The retroperitoneum is not routinely entered during laparotomy and retroperitoneal collections often have non-specific symptoms. Mortality of patients with retroperitoneal abscesses was historically as high as 22%-46% [9], but earlier diagnosis, usually using CT, and management with percutaneous drainage and systemic antibiotics have been instrumental in optimizing patient outcomes and reducing mortality in more recent estimates to 2.7 - 3.4% [10,11]. In this case the premorbid status and severity of the complications undoubtedly contributed to the failure of this post-operative management strategy and the poor outcome [9].

Additionally, in this case there was co-existing acute suppurative appendicitis which we hypothesize occurred secondary to transmural bacterial spread from the inflamed sigmoid colon. Part of the sigmoid colon was located in the right iliac fossa and the appendix was fibrosed to the inflamed proximal sigmoid in the midline. Serosal appendicitis can occur with an extra-appendiceal source of inflammation but in this case histology demonstrated full thickness appendiceal ulceration, presumably a result of the delayed presentation facilitating transmural spread [12].

## Conclusion

This case demonstrates the importance of cross-sectional imaging to diagnose the complications of sigmoid diverticulitis, both pre- and post-operatively, to facilitate appropriate and prompt multi-disciplinary management. Antibiotics, surgical resection, image-guided percutaneous drainage and high-quality supportive care are all important to optimize outcomes. In complicated cases early post-operative imaging is also essential to identify potential complications, particularly when there is a suspicion of retroperitoneal involvement that may not be visualized intra-operatively.
